# Cytomegalovirus (CMV) Infection and Latency

**DOI:** 10.3390/pathogens10030342

**Published:** 2021-03-15

**Authors:** Christine M. O’Connor

**Affiliations:** Genomic Medicine, Lerner Research Institute, Cleveland Clinic, Cleveland, OH 44016, USA; oconnoc6@ccf.org

Cytomegalovirus (CMV) is a herpesvirus that infects a majority of the human population worldwide. While for the most part, latent infection remains asymptomatic in healthy, immune-competent individuals, the virus poses a significant threat to those with weakened immune systems. CMV-associated disease following reactivation of latent infection is a risk factor for transplant patients undergoing immunosuppressive therapies, cancer patients treated with aggressive chemotherapies, immunocompromised AIDS patients, and even otherwise healthy individuals suffering from diseases such as atherosclerosis and inflammatory bowel disease. The full spectrum of CMV-related diseases is still being determined, but it is well-accepted that CMV infections represent a significant and under-addressed medical burden. The current standard of care for antiviral therapies targets the late stages of viral replication, a stage at which the disease is already primed to occur, thus underscoring the need for novel treatments targeting CMV prior to disease onset. This necessitates enhanced understanding of the key biological determinants of the latency and factors that dictate the reactivation phase of infection.

The research articles included in this Pathogens Special Issue highlight the need for multifaceted approaches to better understand latency and reactivation, as well as to provide novel insights to these processes. Included in this collection are research articles that leverage novel in vivo model systems using rodent CMVs, which provide important insights to viral reactivation that extend beyond tissue culture studies. The strength of these systems allows investigators to assess viral dissemination [1], immunological responses [2], and the contribution of infection to the pathologies following transplant [3]—a major advancement to the field. In addition, the review articles in this Special Issue illuminate the important advances made by CMV investigators on the latent and reactivation phases of CMV infection [4,5,6,7]. It is evident from these reviews that the success of these stages of infection rely on the interplay of the host and the virus and are highly complex, involving both viral and cellular factors, many of which have yet to be revealed. I hope the reader finds this collection both timely and compelling, and that the wealth of knowledge provided within provides novel insights to this human pathogen.
